# Gene Expression Profiling in Rodent Models for Schizophrenia

**DOI:** 10.2174/157015910793358132

**Published:** 2010-12

**Authors:** Jessica E. Van Schijndel, Gerard J.M Martens

**Affiliations:** Department of Molecular Animal Physiology, Donders Institute for Brain, Cognition and Behaviour, Centre for Neuroscience & Nijmegen Centre for Molecular Life Sciences (NCMLS), Faculty of Science, Radboud University Nijmegen, 6525 GA, Nijmegen, The Netherlands

**Keywords:** Animal model, behavior, genetics, microarray, mRNA expression, neurodevelopment, pharmacology.

## Abstract

The complex neurodevelopmental disorder schizophrenia is thought to be induced by an interaction between predisposing genes and environmental stressors. In order to get a better insight into the aetiology of this complex disorder, animal models have been developed. In this review, we summarize mRNA expression profiling studies on neurodevelopmental, pharmacological and genetic animal models for schizophrenia. We discuss parallels and contradictions among these studies, and propose strategies for future research.

## INTRODUCTION

Schizophrenia is a complex psychiatric disorder with a lifetime risk of ~1%. The first symptoms usually manifest during late adolescence or early adulthood, but the origin of the disorder is thought to be neurodevelopmental [1, 2]. The disorder is characterized by delusions, hallucinations, disorganized speech, grossly disorganized or catatonic behaviour, and negative symptoms [3]. Negative symptoms concern the absence of or reduction in basic emotional and behavioural processes and include e.g. flat or blunted emotions, poverty of speech, the inability to experience pleasure, and lack of motivation. Although at present not considered a diagnostic criterion, many patients suffer from cognitive impairments such as problems with attention, concentration, learning, memory and abstract thinking [4, 5]. Environmental factors in combination with predisposing genes appear to be important for the aetiology of schizophrenia. Environmental factors include prenatal infections, maternal malnutrition during pregnancy, obstetric complications, poverty, child abuse and neglect, war trauma, loss of parent and migration [5-8]. Such factors may affect gene expression by epigenetic mechanisms [9, 10]. Family, twin and adoption studies have shown that schizophrenia is a disorder with a substantial genetic contribution [11, 12]. On the basis of linkage analyses, loci on 21 of the 23 chromosomes have been linked to the disorder. A meta-analysis has revealed that in various populations chromosomal region 2q, but also 1q, 8p, 22q and other loci, are associated with susceptibility to schizophrenia [13]. Follow-up studies on the regions implicated by the genome scans have resulted in positional candidate genes such as neuregulin-1 (*NRG1*; 8p12) and catechol-O-methyl-transferase (*COMT*; 22q11.21) [14]. However, the final outcome of genetic research on schizophrenia is at present unclear, the search for vulnerability genes for schizophrenia has not resulted in consistent findings, and many genes associated with psychiatric disorders have only a small effect and are mostly linked to more than one disorder [15]. The inconsistent results may arise from the clinical heterogeneity displayed by these disorders [16]. The debate continues on whether in the fifth edition of the Diagnostic and Statistical Manual of Mental Disorders (DSM-V) schizophrenia should be considered as a single disorder or as a group of several disease entities [17].

In order to get a better insight into the function of candidate genes and the aetiology of the disorder in general, multiple animal models have been developed. A problem encountered here is that the most prominent symptoms of schizophrenia - delusions, hallucinations and thought disorder - can not be reproduced in rodents and even not in non-human primates. Furthermore, a well-established genotype, cellular phenotype or other biological marker that is characteristic for the disorder is not available for validation of a model. Nevertheless, a number of symptoms such as e.g. behaviours related to increased dopaminergic transmission (dopamimetric-induced hyperlocomotion), social withdrawal (reduced contact with unfamiliar partners), loss of prepulse inhibition (PPI, a symptom at the interface of psychosis and cognition) and cognitive deficits (impaired performance in a spatial memory test) have been observed in animal models [18, 19]. It is therefore generally acknowledged that animal models may provide new insights into the pathophysiology and aetiology of schizophrenia [18]. In general and based on the way they have been created, animal models can be divided into three groups: (1) neurodevelopmental models, (2) pharmacological models and (3) genetic models. Such animal models for schizophrenia and their characteristics have been described in many excellent papers and reviews [20-22]. This review focuses on microarray mRNA expression data obtained from the schizophrenia animal models (Table **1**).

## mRNA EXPRESSION PROFILING IN NEURODEVELOPMENTAL ANIMAL MODELS

To generate a neurodevelopmental animal model for schizophrenia, environmental factors such as stress and viral infection are employed and since they should affect brain development the factors are applied to animals prenatally (or at a young age). Epidemiologic evidence has suggested that maternal infection by a virus may lead to the genesis of schizophrenia [23, 24]. In mice, prenatal influenza infection causes effects on brain structure and function such as increased serotonin levels [25]. To establish a schizophrenia animal model, C57BL6J mice have been infected on pregnancy day 18 with H1N1 influenza virus. This resulted in a moderate, sublethal infection with a mean virus titer of 10^5.25^ cell culture infectious dose (CCID)_50_/ml [26]. Brain tissues from the offspring of these mice were collected for mRNA expression profiling at post natal day (PND) 0, 14 (childhood) and 56 (adulthood). Analysis with the mouse genome 430 2.0 array (Affymetrix) showed that in the brains of the prenatally exposed mice compared to the brains of control offspring the highest number of genes were up- or downregulated at PND 0 (72, 175 and 157 genes in frontal, hippocampal and cerebellar brain areas, respectively) and PND 56 (110, 62 and 96 genes, respectively), relative to PND 14 (33, 21 and 16, respectively). A number of genes was validated by quantitative polymerase chain reaction (qPCR), including v-erb-B2 avian erythroblastic leukaemia viral oncogene (Erbb4), semaphorin 3A (Sema3a), very-low density lipoprotein receptor (Vldlr), and ATPase,Na+/K+ transporting, beta 2 polypeptide (Atp1b2) [26]. These genes had been implicated previously in the aetiopathology of schizophrenia [27-29]. Compared to viral infection in Balb/c mice at pregnancy day 9 [30], more and other genes were differentially expressed in the offspring of mice infected at pregnancy day 18 [26]. One of the genes differentially expressed in the offspring of mice infected at either pregnancy day 9 or day 18 was *forkhead box P2* (*Foxp2*) [26, 30]. *Foxp2* polymorphisms have been linked to schizophrenic patients with auditory hallucinations [31]. Another viral infection in rats, Neonatal Borna disease (NBD) virus infection, results in life-long viral persistence and causes behavioural and neurodevelopmental abnormalities. In the hippocampus and cerebellum of 4-weeks old Lewis rats that within 12 hours of birth had been inoculated into the right cerebral hemisphere with NBD, gene expression has been analysed with an 8K oligonucleotide array. The expression of metallothionein 1a (*Mt1a* or *MT-I/-II*) was increased ~9- and ~3-fold in the cerebellum and hippocampus, respectively. Furthermore, the expression of *Mt3* was increased in astrocytes and of solute carrier family 30 (zinc transporter) member 3 (*Slc30a3*/*ZnT-3*) was decreased in the hippocampus. The alterations in *ZnT-3* expression, and astrocytic *Mt1a* and *Mt3* induction, in combination with previously described changes in the expression of cytokine- and apoptosis-related products in NBD rats, suggest a multifactorial pathway leading to selective damage [32].

Another environmental factor that has been implicated in the aetiology of schizophrenia is prenatal stress [33]. To create an animal model, repeated variable stress paradigms have been applied to pregnant Sprague–Dawley rats during the last week of gestation. A microarray analysis of the frontal pole brain area of the prenatally stressed adult offspring and non-stressed adult controls revealed significant changes in 35 genes. Three differentially expressed genes encoded neurotransmitter receptor subunits or associated proteins, two were involved in calcium/calmodulin signaling, two were associated with neurotransmitter vesicles or vesicle recycling and two were Na^+^/K^+^-transporting ATPases [34]. Both the neurodevelopmental animal model based on prenatal influenza infection [26] and the model generated by prenatal stress [34] displayed differential expression of Na^+^/K^+^-ATPase genes and potassium voltage-gated channel genes. However, the majority of the differentially expressed genes was differentially expressed in only a single animal model. 

A third neurodevelopmental animal model constitutes the neonatal ventral-hippocampal (VH) lesion rat model that exhibits many of the behavioural features of pharmacological schizophrenia models [18, 35, 36]. The locomotor abnormalities induced by the VH lesions differ between rat strains and therefore transcript expression was compared between saline- or haloperidol-treated adult VH-lesioned Fischer-344 and Lewis rats [37]. Three variables were compared, namely with respect to strain (Fischer-344 versus Lewis), to age (day 6 versus day 70) and to treatment (saline versus haloperidol), and suppression subtraction hybridization was used to identify changes in transcript levels. Genes differentially expressed in all three cases included mitochondrial cytochrome oxidase (complex IV) subunits including COX1 and 2, mitochondrial tRNA, g and z isoforms of 14-3-3, adenosine monophosphate deaminase 3 (*Ampd3*), and a 25-kD synaptosomal associated protein (*SNAP-25*) [37]. 

A further neurodevelopmental animal model concerns the maternal deprivation rat model. Maltreatment, such as abuse and neglect, is a susceptibility factor for psychiatric disorders [38]. Fisher 344 rat pups were separated for 8 hours from their mother every other day between PND 2 and 10. At the age of 13 weeks, the rats were sacrificed and gene expression in hippocampal slices was analysed with an U34A (Affymetrix) array. The expression of fifteen genes was upregulated and of nine genes downregulated. The most prominent change in expression was found for the transthyretin (*Ttr*) gene. In the brain the expression of this gene is nearly restricted to the choroid plexus and therefore the authors speculated about a possible function for the choroid plexus in stress [39]. However, in other studies the differential expression of *Ttr* was considered to be the result of a tissue-isolation artefact (contaminating choroid plexus) rather than being a differentially expressed gene of interest [40].

Yet another example of a neurodevelopmental animal model are three-weeks old, weaned Institute for Cancer Research mice that were isolated for 3 days, 2 weeks or 4 weeks, a treatment that may also affect brain development [41]. In these mice, mRNA expression in the dentate gyrus was analysed with a GeneChip mouse genome 430 2.0 array (Affymetrix), but no obvious changes were found. However, using a general linear model incorporating the feeding period the downregulation of two genes was observed, namely of nuclear receptor subfamily 4, group A, member 2 (*Nr4a2*/*Nurr1*) and neuronal PAS domain protein 4 (*Npas4*) [42].

## mRNA EXPRESSION PROFILING IN PHARMACOLOGICAL ANIMAL MODELS

The neuropharmacological animal models for schizophrenia are based on specific neurotransmitter systems (typically dopamine (DA) and glutamate). Psychotomimetic substances (DA agonists, N-methyl-D-aspartate (NMDA) antagonists) have been used to generate schizophrenia-like symptoms in animals. In rodents, phencyclidine (PCP) and other antagonists of the NMDA-type glutamate receptor produce positive and negative symptoms, and cognitive disturbances remarkably similar to those observed in schizophrenia patients [43]. Animals in which these symptoms were induced by PCP or related compounds have therefore been considered to represent useful pharmacological models for this complex disorder. A single administration of PCP during the late foetal or early postnatal period in rats (corresponding to the third trimester of human pregnancy) produced behavioral symptoms indicative of NMDA receptor blockade lasting for 8 to 10 hours and resulted in increased neuronal death by apoptosis [44]. Acute and chronic prenatal or neonatal administration of PCP to rats led to changes in behaviour of the adult animal, such as hyperlocomotion, impairments in information processing and increased stereotypic behaviour, and changes in the glutamatergic, dopaminergic and GABAergic systems [reviewed in 43]. The influence of PCP administration on mRNA expression levels has been reported already in 2002. Applying a Clontech Atlas glass mouse 1.0 arrays, 28 genes have been found to be upregulated (including tyrosine 3-hydroxylase, *Th*) and 45 genes were downregulated (including voltage-gated potassium channel, subfamily H (eag-related), member 1, *Kcnh1*) in mice injected with PCP once a day for 24 days [45]. Using newer microarrays representing more genes and following acute PCP treatment, mRNA expression changes in 477 genes have been observed in adult rat prefrontal cortex (PFC), a brain area associated with cognitive dysfunction in schizophrenia. One of the downregulated genes was voltage-gated K^+^ channel *Kcna4 *[46], yet another potassium voltage-gated channel gene differentially expressed in an animal model for schizophrenia. The observed upregulation of *c-fos*, *Klf2*, *Nr4a3*, *Klf4* has been confirmed in an independent study that also reported the upregulation of Leiomodin 2 (*Lmod2*) in the thalamus of male rats following acute PCP administration at PND 50 but not at PND 8, suggesting that *Lmod2* may be involved in the age-dependent onset of drug-induced schizophrenia-like psychosis [47].

Another antagonist of the NMDA receptor is ketamine, a PCP derivative [48]. In the to our knowledge only report on the genome-wide analysis of mRNA expression following ketamine administration, 50 genes have been found to be differentially expressed in the combined hippocampus, amygdala and hypothalamus regions of singly injected mice [49]. A number of the differentially expressed genes is associated with tissue/organ/system development, such as gastrulation brain homeobox 2 (*GBX2*) and early growth response 2 (*EGR2*) [50,  51], or cancer/apoptosis, such as protein kinase C delta (*PRKCD*), secreted phosphoprotein 1 (*SPP1/OPN*), prostaglandin-endoperoxide synthase 2 (*Ptgs2; *also named cyclo-oxygenase 2, *COX-2*) and growth arrest and DNA-damage-inducible 45 gamma (*GADD45g*) [52-55]. The investigators followed up on Troponin T1 (*Tnnt1*) since this gene showed consistently strongly (2- to 4-fold) elevated expression in the brains of treated mice and was found to be exclusively regulated by FoxO1, a gene thought to be involved in metabolic pathways and energy homeostasis [49]. The activation of *FoxO1* in the hypothalamus of normal mice increases food intake and body weight, whereas its inhibition decreases both processes, suggesting that *FoxO1* indeed plays a role in central energy homeostasis and hunger/appetite behaviours [56]. Weight gain and abdominal adiposity may be central to the pathophysiology of the metabolic syndrome observed among neuropsychiatric patients [57], and *Tnnt1* may be the link between ketamine and weight gain [49]. 

A further antagonist of the NMDA receptor is MK-801 or dizocilpine [58]. The effect of MK-801 on mRNA expression levels following spinal cord injury (SCI) has already been analysed in 2002 [59]. Although SCI is not linked to schizophrenia, it is still of interest to compare the effects of the NMDA receptor antagonists PCP, ketamine, and MK-801 following SCI. One hour after traumatizing adult male Sprague-Dawley rats, the mRNA levels of 165 genes and expressed sequence tags (ESTs) were changed on rat neurobiology U34 microarrays (Affymetrix). The affected mRNA levels were mostly of genes that are involved in transcription, inflammation, cell survival and membrane excitability. Intrathecal administration of MK-801 directly to the injury site at 1 hour before the injury and at 1 hour after the injury reversed the effect of SCI on the expression levels of about half of the SCI-affected mRNAs. Additionally, MK-801 treatment changed the mRNA levels of 168 genes and ESTs that had not been affected by SCI [59]. In another study, ischemic insults were induced in fasted male Wistar rats and hippocampal mRNA levels were analysed with an Affymetrix Rat Genome 230 2.0 array (31020 probe sets). Directly following the lethal ischemic insult 967 genes were upregulated in vehicle/IPC versus MK-801/IPC rat brains, while 926 genes were downregulated. The investigators further investigated the downregulation of *COX-2* (*Ptgs2) *and confirmed by Western blot analysis that at 24 h following lethal ischemia the expression of *COX-2/Ptgs2* was substantially reduced in the vehicle/IPC group in comparison to the vehicle/sham (non-preconditioned) and MK801/IPC groups [60]. Thus, in this study MK-801 treatment increased the expression of *COX-2/Ptgs2 *while in the study of Lowe *et al.* (2007) ketamine treatment reduced the expression of this gene [49, 60]. Since the two studies were published simultaneously, this discrepancy was not discussed by the authors. The use of different conditions (3 mg/kg MK-801 prior to IPC, followed by lethal ischemia after 72 hours *versus* a single i.p. injection of 80 mg/kg ketamine) may explain the discrepancy. In another study, the influences of MK-801 and the uncompetitive NMDA receptor antagonist memantine (1-amino-3, 5-dimethyladamantane) on brain mRNA expression were compared [61]. Male adult Wistar rats were i.p. injected with 5-50 mg/kg memantine or 1 mg/kg MK-801 and the posterior cingulate and anterior retrosplenial cortices were dissected for RNA isolation. Microarray analyses (1090 genes) revealed that 34 genes were regulated by the MK-801 treatment and that 28 genes were differentially expressed by the memantine treatment. Of these, 13 genes were regulated by both treatments, including nuclear factor of kappa light polypeptide gene enhancer in B-cells inhibitor, alpha (*Nfkbia*)*, *neurotrophic tyrosine kinase, receptor, type 2 (*Trk2*)*,* heat shock protein 4 (*Hspa4/Hsp70*)*,* heat shock protein 5 (*Hspa5/GRP78*)*, *GDP dissociation inhibitor 1 (*Gdi1*)*, *hippocalcin-like 4 (*Hpcal4/NVP-2*), glutamate receptor, ionotropic, N-methyl D-aspartate 2B (*Grin2b/NMDAR2B*)*, * Neuromedin B transporter (*Nmbr*)*, *neuropeptide Y (*Npy*)*,* corticotropin releasing hormone (*Crh/CRF*), Na^+^/K^+^ transporting ATPase 2β (*Atp1b2*), Ca^2+^ transporting ATPase-isoform 2 (*Atp2b2*) and protein phosphatase 2 (formerly 2A), catalytic subunit, beta isoform (*Ppp2cb*) [61]. In a related study, male Wistar rats were repeatedly i.p. injected with 0.13 mg/kg MK-801 once daily for 14 days. Gene expression in the bilateral amygdalae was analysed with a Rat Expression Array 230A Array spotted with 15923 probe sets (Affymetrix). Twenty-three genes were downregulated and sixteen genes were upregulated of which arginine vasopressin (*Avp*), prostaglandin D2 synthase (*Ptgds*), myelin-associated oligodendrocyte basic protein (*Mobp*; all downregulated) and insulin-like growth factor binding protein 2 (*Igfbp2*)*,* somatostatin receptor 2 (*Sstr2*)*,* ectonucleotide pyrophosphatase/phosphodiesterase 2 (*Enpp2*) and *Ttr* (upregulated) were confirmed by qPCR [62]. In the four studies applying MK-801 treatment [59-62], four different sets of affected genes have been identified, presumably due to the differences in the use of animals, injection methods, brain tissues and arrays. The significance of these four gene sets remains to be established.

The most common hypothesis concerning the aetiology of schizophrenia is the dopaminergic hypothesis which was built on the finding that the clinical effectiveness of antipsychotic drugs is related to their affinity for DA receptors. Since then advances in neurochemical imaging and a further understanding of the genetic aetiology of schizophrenia have led to the hypothesis that multiple (genetic, environmental) factors interact, resulting in DA dysregulation in schizophrenia patients [63]. It is therefore not surprising that DA receptor agonists are useful in creating animal models to explore this hypothesis. An example of a DA receptor agonist is apomorphine [64]. Apomorphine-susceptible rats (so-called APO-SUS rats) constitute a model for schizophrenia. These rats have been pharmacogenetically selected based on their susceptibility for apomorphine as measured by their gnawing behaviour (>500 gnawing counts in 45 minutes). Extensive characterization of the APO-SUS rats and their phenotypic counterparts apomorphine-unsusceptible (APO-UNSUS) rats has shown that both genetic and environmental factors influence the complex phenotype of these rats [65]. Microarray analysis of hippocampal mRNA using Rat NeuroGEM 2.02 microarrays containing 8478 Sprague Dawley rat nervous system cDNAs revealed that only one gene was differentially expressed between the two rat lines, namely *Aph-1b*. Further analysis showed that the expression difference of this γ -secretase component was caused by a gene-dosage imbalance, namely one or two copies of *Aph-1b* in APO-SUS rats and three copies in APO-UNSUS rats [40].

Pharmacological models can also be generated by the treatment of animals with schizophrenia drugs such as olanzapine or clozapine [66]. Olanzapine is a second-generation antipsychotic agent that exhibits greater 5HT2A than D2 receptor antagonism and has high affinity for a number of other receptors, such as muscarinic, α1-adrenergic, histamine-H1, 5HT2A, 5HT2C, 5HT1A, 5HT6, 5HT7, and 5HT3 receptors [67]. Additionally, olanzapine facilitates NMDA neurotransmission *via* its effects on glutamatergic NMDA and AMPA receptors [68]. To study the influence of chronic olanzapine treatment, adult male Sprague–Dawley albino rats were administered daily for 21 days with 1 ml olanzapine (2 mg/kg/day, i.p.) and sacrificed 24 h after the last injection under deep anaesthesia. Gene expression in the frontal cortex was analysed using an Affymetrix rat genome 230 2.0 array set. The results showed downregulation of 31 genes and upregulation of 38 genes in the drug-treated *versus *control brains. The application of real-time qPCR verified the magnitude and direction of the changes for a number of significantly affected genes, including *calbindin 3 (S100g)*, *reelin*, *RGS2*, and *insulin 2* [69]. Recently, Duncan *et al.* (2008) compared the effects of the conventional antipsychotic drug haloperidol on gene expression with the effects of the atypical drugs olanzapine and clozapine [70]. Haloperidol (1 mg/kg), olanzapine (10 mg/kg), or clozapine (10 mg/kg) was administered *via* daily i.p. injection for 7 days to male C57BL/6 mice. Five hours after the last injection, the mice were sacrificed under 4% halothane anaesthesia. Whole-brain gene expression profiles were analysed using the Affymetrix GeneChip Mouse Genome 430 2.0 microarrays. Data analysis revealed that ~12,000 out of over 39,000 transcripts showed > 1.5-fold change by at least one drug compared with saline-treated controls and 249 transcripts were coregulated by all three antipsychotic drugs. Twenty candidate genes were chosen for further expression analysis based on their regulation by multiple drugs, the chromosomal location of their human orthologs, pattern of expression, or relevant neurobiological function [70]. Some of these genes have been genetically associated with schizophrenia, namely neuroplastin (*Nptn/Sdfr1*), numb homolog (Drosophila)-like (*Numbl*)*, *proteolipid protein 1 (*Plp1*)*, *gamma-aminobutyric acid (GABA) A receptor, α1 (*Gabra1*)*, *and reticulon 4 (*Rtn4/(NogoA*) [71-75]. Thirteen out of the twenty genes showed a statistically significant change in mRNA expression as determined by qPCR. To examine whether the changes in mRNA expression had led to changes into protein expression, whole-brain expression of three of the proteins was analysed with Western blots. The Kv channel interacting protein 3 (Kcnip3) was downregulated in brain tissues of the haloperidol- and olanzapine-treated rats, K_v_1.1 was upregulated in the haloperidol-treated rats and NEDD4 was downregulated in the olanzapine-treated rats. The authors hypothesised that the specific effects of multiple antipsychotic drugs on the expression of four genes regulating voltage-gated ion channels –potassium voltage-gated channel, Shaker-related subfamily, member 1 (*Kcna1*), Potassium voltage-gated channel, Shaker-related subfamily, beta member 1 (*Kcnab1*), *Kcnip3* and Neural precursor cell expressed, developmentally downregulated gene 4 (*Nedd4*)– may indicate a role for these neuronal regulators in antipsychotic drug action [70]. In another study on clozapine-treated rats, no genes encoding a potassium voltage-gated ion channel have been found to be deregulated [76]. Male Wistar rats (~200 g) were given a single clozapine (25 mg/kg) i.p. injection and sacrificed 1, 6 or 24 hrs after the drug administration. Gene expression in the prefrontal and anterior cingulate cortices was analyzed with an Atlas Rat 1.2 Array filter (Clontech) of 1176 sequence-verified cDNAs. *In situ* hybridization was used to validate the regulation of 35 genes with the most pronounced changes in the filter array experiments or their close functional relatives. These genes belong to many different functional classes. A modest but consistent increase (~15%) of chromogranin A (Chga) mRNA was observed in the outer and inner layers of the prefrontal cortex at 6 hrs after a single clozapine injection. Twenty four hrs after clozapine treatment synaptotagmin V mRNA was downregulated in the inner layers of the frontal cortex (~30%), and in the outer and inner layers of the parietal cortex (~15–20%). These results were consistent          with the filter array results. *In situ* hybridization confirmed the upregulation (~20%) of calcineurin A mRNA in the inner layers of the infralimbic cortex 6 hrs after clozapine treatment and a more pronounced upregulation (~70%) in the inner layers of the frontal cortex 24 hrs after the treatment. Following a chronic treatment (daily injection for 17 days) with haloperidol or clozapine, six of the 35 genes were differentially expressed as shown by *in situ* hybridization. The chronic clozapine treatment changed the expression of *Chga*, Son of sevenless (*Sos*) and Syntaxin binding protein 1 (*Stxbp1*/*Sec1*) [76]. An effect of such chronic clozapine treatment has been reported previously for Chga mRNA expression in striatum, nucleus accumbens, dorsal raphe nucleus and prefrontal cortex [77, 78]. The expression of Inhibitor of DNA-binding 2 (*Id2*), RAB12, member RAS oncogene family (*Rab12*)  and Visinin-like protein 2 was changed by chronic treatment with haloperidol. Interestingly, yisinin-like protein 1 has been found previously to be downregulated 1 and 24 hrs after a single PCP injection (8 mg/kg) in the entorhinal cortex and nucleus accumbens of male Sprague-Dawley rats, respectively [79]. Investigators who studied previously the effect of PCP on gene expression in mice [45] also studied gene expression in male Institute of Cancer Research mice (Charles River Laboratories) that received daily subcutaneous injections of clozapine (7.5 mg/kg) for 20 days. Mice were sacrificed 20 hrs after the last injection and mRNA expression in the cerebral cortical tissues was analysed with a Clontech Atlas Mouse 1.2 Array membrane. Of the 1176 genes on this membrane, the expression of potassium channel *Kcnab1* and prostaglandin D2 synthase was decreased, while the expression of serotonin receptor type 2 and myelin-associated oligodendrocytic basic protein was increased [80]. In a more recent study, the chronic effects of haloperidol, clozapine and phenylpropanolamine (an anorexiant drug used to encourage weight loss) on 10-12 week old male C57BL6 mice were examined [81]. Mice were administered with the drugs orally *via* food pellets daily for 31 days and gene expression in the cortices was analysed using the Murine Genome U74A Array (Affymetrix) containing ~12000 genes. The authors focused on differentially expressed genes involved in body weight regulation since antipsychotic drugs such as clozapine are associated with clinically significant weight gain [82]. However, although the mice treated with clozapine had the highest food intake they weighed significantly less than the control mice, presumably because the induced weight gain in rodents treated with antipsychotic drugs is influenced by a variety of factors [83]. The genes aldolase 3 (*Aldo3*) and phospholipase C beta 1 (*Plcb1*), involved in glycolysis and lipid metabolism, respectively, were differentially expressed in the clozapine-treated but not in the haloperidol-treated mice [81], suggesting that these genes are involved in the regulation of body weight rather than in schizophrenia. Although drugs such as haloperidol and the atypical antipsychotic risperidone may have different receptor affinities, they interfere with neural plasticity and signal transduction in a similar manner [84]. Adult Sprague-Dawley rats were daily injected i.p. with haloperidol or risperidone and following acute or chronic (4 weeks) treatment gene expression in the prefrontal/fronto-parieto-temporal cortices was analysed with arrays containing 8000 rat genes. The acute treatment with risperidone affected the expression of more genes than the acute haloperidol treatment (89 and 28 genes, respectively). This could be due to the fact that the high-potential D2-, 5HT2- and NA-antagonist risperidone binds to diverse receptors, while haloperidol acts only as a D2-antagonist. Of the 28 genes differentially expressed upon haloperidol treatment, 18 were also differentially expressed following risperidone treatment. In general, each of the drugs affected genes with specific functions in signal transduction, transcriptional and translational regulation, protein turnover, and cellular metabolism [84]. The effect of chronic risperidone treatment of rats on mRNA expression levels in the frontal cortex was also studied by Chen and Chen [85]. The use of GeneMap Rat Clone Arrays containing cDNA fragments of 1536 genes revealed 17 genes to be differentially expressed, eight of which were validated by qPCR. None of these eight genes were found to be differentially expressed in the acute and chronic risperidone study of Fehér *et al.* [84].

## mRNA EXPRESSION PROFILING IN GENETIC ANIMAL MODELS

In most cases, a genetic animal model for schizophrenia carries a mutation in a gene involved in brain development [86]. The mutation is usually in a dopaminergic or a glutamatergic gene, in line with the use of DA agonists and NMDA antagonists in pharmacological models for schizophrenia [87]. Examples of dopaminergic and glutamatergic mutant animals include the DA transporter (*DAT*), *Nurr1* and glutamate receptor, ionotropic, NMDA2A (*Grin2a*/*NR2A*) knockout (KO) mice, and glutamate receptor, ionotropic, NMDA1 (*Grin1*/*NR1*) knock down mice [88-90]. To our knowledge, gene expression has not been analyzed in any of these nor in other dopaminergic or glutamatergic mutant mice, except in the *Nurr1 *KO mice. *Nurr1*, an orphan member of the steroid/thyroid hormone receptor family, is an absolute requirement for the proper development of midbrain DA neurons. Homozygous *Nurr1 *KO mice fail to generate midbrain DA neurons, are hypoactive and die soon after birth [91]. Gene expression has been analyzed in the ventral midbrain of homozygous *Nurr1* KO mice at embryonic day 12.5 and PND0 using Affymetrix MGU74Av2 and MGU74Bv2 gene chips. Among the differentially expressed genes were DA-related genes such as tyrosine hydroxylase (*TH*) and *DAT*, and also guanosine 5’-triphospate (GTP) cyclohydrolase 1 (*Gch1*/*GTPCH*), a rate-determining enzyme in the synthesis of tetrahydrobiopterin (BH4), a cofactor essential for TH [92].

Genetic animal models have also been developed on the basis of genes thought to play an important role in the aetiology of schizophrenia. One of such genes is Reelin (*RELN*) [93-95] and therefore the heterozygous reeler mouse, with a reelin insufficiency, has become of interest as an animal model [96]. In the reeler mouse brain, the expression of the mouse homologue of Strawberry Notch (*Sno*) was reduced. *In vitro* assays have shown that reelin treatment indeed enhanced the expression of *Sno*. Being a component of the notch signaling pathway in Drosophila [97], *Sno* thus links the reelin signal to the regulation of neurogenesis [98]. A further schizophrenia animal model is represented by the Df1/+ mouse because it carries a hemizygous deletion from *Es2* to *Ufd1l *(referred to as Df1 [99]), a region syntenic with a human 22q11 deletion causing DiGeorge syndrome; 35% of the DiGeorge syndrome patients develop schizophrenia [100]. In hippocampal dentate granule neurons of 10-week-old Df1/+ mice, two genes with a ribosomal protein function (*Rps2* and *Arbp*) have been found to be upregulated. Eleven genes were downregulated, ten of which were located in the 22q11 region, including the gene encoding COMT. The only downregulated gene not located in the deleted region was B-cell CLL/lymphoma 7a (*Bcl7a*), a gene with an unknown function [100]. Other genetic animal models for schizophrenia concern mice lacking the protein kinase C interacting protein/histidine triad nucleotide binding protein 1 (*PKCI/HINT1*) [101, 102], brain-derived neurotrophic factor (*BDNF*) [103-105], neural cell adhesion molecule (*Ncam*) 180 [106], cyclin-dependent kinase 5 (*Cdk5*) [107], neural PAS domain protein 3 (*NPAS3*) [108] or LIM homeobox protein 5 (*Lhx5*) [109], mice carrying a mutation in proline dehydrogenase 2 (*PRODH2*) [110], Disrupted-in-Schizophrenia-1 (*DISC1*) [111, 112] or neuregulin-1 [113] as well as the Chakragati (*Ckr*) mouse [114]. No gene expression analyses have been performed on any of these genetic animal models.

## CONCLUSIONS AND FUTURE DIRECTIONS

Our overview of the microarray analyses performed in animal models for schizophrenia illustrates that we still can not provide a conclusive answer to the question which genes play a role in the aetiology of this complex disorder. The numerous mRNA expression studies have resulted in even more genes that could play a role in the onset of schizophrenia than was previously thought. However, since most of these genes have been found in only a single study, their significance is at present unclear. An exception may constitute the family of sodium and potassium voltage-gated channels that seems of interest as in seven out of the twenty-six microarray studies discussed here at least one member of this family has been found to be differentially expressed [34, 45, 46, 61, 69, 70, 80]. Intriguingly, genes encoding potassium channels have already been implicated in the aetiology of schizophrenia, such as *KCNN3* [115] and recently *KCNH2 *[116]. In particular *KCNH2* appears to be a strong contributor to the schizophrenia phenotype because of the presence of disease-associated SNPs in its gene together with compelling, converging evidence from (functional) brain imaging, mRNA expression and electrophysiological studies [117]*.*

The neurodevelopmental, pharmacological and genetic animal models for schizophrenia have been created in different ways. This circumstance may explain the near lack of overlap between the genes found to be differentially expressed in the various animal models. Moreover, heterogeneity such as the ages of the animals and the ways of treating or sacrificing the animals occurs even within one group of animal models (either neurodevelopmental, pharmacological or genetic). For instance, in the various studies on the pharmacological models the animals were treated differently in that substances were administered at different concentrations and at different time intervals, and the time points at which the animals were sacrificed following drug administration were not the same. Furthermore, before being sacrificed in some of these studies the animals were anaesthetized, while it is known that anaesthesia may well affect gene expression levels [118-121]. In addition, the methods of dissecting the brain areas differed and a number of brain regions were analysed, with each region having its specific mRNA expression pattern. For the gene expression analyses, a variety of microarrays and platforms have been used, ranging in their coverage from a couple of thousand genes to the nearly complete genome. The variation among the various experiments was further increased by the use of different statistical methods and cut-off values. The studies performed with the early microarrays and dealing with only a limited number of genes should therefore be repeated with the newest microarrays or deep sequencing approaches and using the latest knowledge, e.g. regarding the effect of anaesthesia on gene expression. More overlap in the results of the future genome-wide mRNA expression studies may then occur, possibly leading to the identification of common molecular pathways that may well play a role in the aetiology of schizophrenia. At present however the studies performed thus far do not provide decisive insights. Clearly, more molecular analyses of the various animal models for schizophrenia are necessary. It would be of interest to include in such studies new genome-wide technologies to explore, besides mRNA expression profiles, epigenetic (e.g. DNA methylation or histone acetylation), and noncoding RNA and (phospho) protein expression patterns as well.

## Figures and Tables

**Table 1:** An Overview of Schizophrenia Animal Models Used in Microarray Studies

Animal Model	Approach	Number of Gene(s) with Affected mRNA Expression	Ref.
C57BL6J mice infected with H1N1 influenza virus on pregnancy day 18	Mouse genome 430 2.0 array (Affymetrix) covering >39000 transcripts	Hippocampus: 175 (PND0), 21 (PND14) and 62 (PND56) Cerebellum: 157 (PND0), 16 (PND14) and 96 (PND56) Prefrontal cortex: 72 (PND0), 33 (PND14) and 110 (PND56)	[26]
Balb/c mice infected with H1N1 influenza virus on pregnancy day 9	Murine 430 high-density oligonucleotide array (Affymetrix) covering ~20000 mouse genes and ESTs	Whole brain: 39 genes	[30]
Lewis rat pups inoculated with NBD within 12 hours of birth, sacrificed at 4 weeks of age	3DNA Array 350 Expression Array (Genisphere) covering 8064 genes	Cerebellum: MT-I/-II (Mt1a) 9.09 fold increase Hippocampus: MT-I/-II (Mt1a) 2.95 fold increase	[32]
Pregnant Sprague-Dawley rats subjected to a repeated variable stress paradigm from ED14 to ED22, male pups were subjected to acute stress at PND56 and sacrificed	Affymetrix RG U34A microarray covering 8799 genes	Frontal pole: 35 genes	[34]
VH-lesioned Fisher 344 (F344) rats, daily i.p. haloperidol treatment for 14 days starting at PND55	GeneFilter^®^ DNA microarray blots (Research Genetics Inc., Huntsville) covering 37575 cDNA clones	VH-lesioned F344 versus sham-lesioned F344 rats - Frontal tissue: 828 genes Temporal tissue: 768 genes Haloperidol treated *versus* control VH-lesioned F344 - Frontal tissue: 404 genes- Temporal tissue: 595 genes	[37]
New born F344 rats separated from their dams for 8 hours every other day (PND2 to 10)	Affymetrix rat U34A microarray	Hippocampal slices: 24 genes including *Ttr* (4.85 fold decrease)	[39]
Institute for Cancer Research (ICR) Mice subjected to isolated rearing for 4 weeks	GeneChip mouse genome 430 2.0 Array (Affymetrix) covering 45101 probe sets	Dentate gyrus: 22 genes including *Nr4a2/Nurr1* and *Npas4*	[42]
ICR mice, daily i.p. PCP for 24 days	Clontech Atlas glass array mouse 1.0 covering 1000 cDNAs	Cerebral cortical tissue: 73 genes	[45]
Male Sprague-Dawley rats injected once with PCP sacrificed 4 hours after injection	Affymetrix rat U34A microarray covering 8784 probe sets	Prefrontal cortex: 477 genes	[46]
Male Wistar rats injected with PCP at PND8 or PND50 and sacrificed 60-90 minutes after injection	Affymetrix rat genome 230 2.0 array covering over 30000 transcripts	Thalamus: *c-fos, Klf2, Nr4a3, Klf4* and leiomodin2 (*Lmod2*)-like	[47]
Sprague-Dawley rats with a spinal cord (segment T8) injury administered with MK-801 one hour before and after the injury	Affymetrix Rat Neurobiology U34 oligonucleotide microarray containing 1322 probes	combined T7,T8 and T9 spinal cord segments: 168 genes that were not affected by spinal cord injury itself	[59]
B6C3F1 mice treated with a single i.p. s(+)ketamine injection anaesthetized and sacrificed 30 minutes post injection	Affymetrix MGU-74 av2 GeneChips^®^ covering 12488 genes	Hippocampus/amygdala/hypothalamus: 50 genes	[49]
Male Wistar rats (injected with MK-801) were subjected to ischemic preconditioning (IPC) by sublethal bilateral carotid artery occlusion (BCAO) and 3 days later to lethal BCAO	Affymetrix RG 230 2.0 array covering 31020 probe sets	Hippocampus: 1893 genes	[60]
Male Wistar rats i.p. injected with MK-801 or memantine narcotized with CO_2_ and sacrificed 4 hours post injection	Rat 1.0 Atlas Glass Microarrays (Clontech) containing 86 controls and 1090 well characterized transcripts	Posterior cingulate and anterior retrosplenial cortices: MK-801: 34 genes Memantine: 28 genes	[61]
Male Wistar rats i.p. injected with (+)-MK801 maleate daily for 14 days, tested for social interaction and sacrificed on day of last injection	GeneChip^®^ Rat Expression 230A array (Affymetrix) containing 15923 probe sets	Amygdala: 39 genes including *AVP* and *Ttr*	[62]
APO-SUS *versus* APO-UNSUS Wistar rats	Rat NeuroGEM 2.02 microarrays containing 8478 Sprague Dawley rat nervous system cDNAs	Hippocampus: 1 gene, *Aph-1b*	[40]
Male Sprague–Dawley albino rats administered i.p. daily with olanzapine and sacrificed 24 h after the last injection under deep anaesthesia	Affymetrix rat genome 230 2.0 array set containing ~28000 rat genes and ESTs	Frontal cortex: 69 genes	[69]
Male C57BL/6 mice i.p. injected with haloperidol, olanzapine, or clozapine daily for 7 days and sacrificed under 4% halothane anaesthesia five hours post injection	Affymetrix GeneChip Mouse Genome 430 2.0 microarrays containing over 39000 transcripts	Whole brain:11941 transcripts by one or more drugs, 249 transcripts by all 3 drugs	[70]
Male Wistar rats injected i.p. with clozapine and sacrificed 1, 6 or 24 hrs post injection	Atlas Rat 1.2 Array filter (Clontech) covering 1176 sequence-verified cDNAs	Anterior cingulate and prefrontal cortices: 217 genes including 22 genes with a presynaptic function	[76]
ICR mice injected subcutaneous with clozapine daily for 20 days and sacrificed 20 hours post injection	Atlas Rat 1.2 Array filter (Clontech) covering 1176 sequence-verified cDNAs	Cerebral cortical tissues: undefined number of genes including potassium channel, prostaglandin D2 synthase, serotonin receptor type 2 and myelin-associated oligodendrocytic basic protein	[80]
C57BL6 mice received daily for 31 days haloperidol or clozapine or phenylpropanolamine via food pellets	Murine Genome U74A Array (Affymetrix) containing ~12000 genes	Cortex: 1156 transcripts in one or more models	[81]
Sprague–Dawley rats injected i.p. daily with Haloperidol or Risperidone and sacrificed 96 hours (acute treatment) or 4 weeks (chronic treatment) after initiation of drug treatment.	PXM oligo-nucleotide slides (Full Moon Biosystems) containing 8000 rat gene-specific, amino-modified oligonucleotides	Prefrontal/fronto-parieto-temporal cortices: Haloperidol:- acute: 28 genes - chronic: 9 genes Risperidone: - acute: 89 genes - chronic: 17 genes	[84]
Male Sprague-Dawley rats injected i.p. with risperidone daily for 4 weeks and sacrificed under CO_2_ anesthesia 24 hours post injection	GeneMap Rat Clone Arrays (Genomic Solutions Inc.) containing cDNA fragments covering 1536 genes	Frontal cortex: 17 genes	[85]
*Nurr1* KO mice generated by mating of *Nurr1* +/- animals. Pregnant females were sacrificed at ED12.5 and Nurr1 KO embryos selected	Affymetrix MGU74Av2 and MGU74Bv2 gene chips covering	Ventral Midbrain: Undefined small number of genes including *GTPCH*	[92]
Reeler mouse at ED11.5	Qiagen 70mer oligo chip	Brain: 1 gene, mouse *Strawberry Notch 1 mSno1*	[98]
Ten weeks old DfI/+ mice	Agilent Oligo Mouse Microarrays containing 60-mer oligonucleotide probes for 20915 murine genes	Hippocampal dentate granule neurons: 13 genes	[100]
